# Rapid Improvement in Weight, Body Composition, and Glucose Variability With Semaglutide in Type 1 Diabetes

**DOI:** 10.7759/cureus.61577

**Published:** 2024-06-03

**Authors:** Hoda Gad, Rayaz A Malik

**Affiliations:** 1 Research Department, Weill Cornell Medicine-Qatar, Doha, QAT; 2 Institute of Cardiovascular Medicine, University of Manchester, Manchester, GBR

**Keywords:** type 1 diabetes mellitus (t1dm), continuous glucose monitoring (cgm), glucose variability, semaglutide, glucagon-like peptide-1 receptor agonist

## Abstract

The efficacy of glucagon-like peptide-1 receptor agonists (GLP1-RA) in type 2 diabetes mellitus is well-established. GLP1-RAs are not approved for use in type 1 diabetes mellitus (T1DM). A 34-year-old woman with a 23-year history of T1DM presented for review for weight gain (weight 63 kg, BMI 26.9 kg/m^2^) and increased HbA1c (8.3%) and glycemic variability. Subcutaneous semaglutide (1 mg weekly) was commenced. After two months, there was decrease in weight by 12 kg, body fat percent by 15%, visceral fat by 7%, and a reduction in insulin dose, glycemic variability, and HbA1c. Semaglutide could be an important adjunct to insulin treatment in T1DM.

## Introduction

The efficacy of glucagon-like peptide-1 receptor agonists (GLP1-RA) in type 2 diabetes mellitus is well-established with weight loss, improved glycemic control without hypoglycemia [1], and an improvement in cardiovascular outcomes [2,3]. GLP1-RAs are not approved for type 1 diabetes mellitus (T1DM) and may require caution in patients with high glucose variability and an increased risk of hypoglycemia. A recent case report of an obese woman with T1DM treated for six months with semaglutide showed favorable outcomes with a reduction in insulin dose and weight and improvement in glycemic variability [4].

## Case presentation

A 34-year-old woman with T1DM for 23 years presented with weight gain (~10 kg) over three to four years and increased glycemic variability. She was taking multiple daily insulin injections of 14 U insulin glargine 300 units/mL daily and insulin Lispro 100 units/mL using carb counting with a carbohydrate-to-insulin ratio (CIR) of 10:1 and correction doses as needed when the 2-hour postprandial blood glucose was >9.99 mmol/L (180 mg/dL) with an insulin sensitivity factor (ISF) = 3 units for each 5.55 mmol/L (100 mg/dL) above target. Continuous glucose monitoring (CGM) was performed using the Freestyle Libre 2 system.

Assessment

Her weight was 63 kg and body mass index (BMI) was 26.9 kg/m^2^. Her cardiometabolic panel was as follows: creatinine 0.71 mg/dL, estimated glomerular filtration rate (eGFR) >90 mL/min/1.73m^2^, bilirubin 0.23 mg/dL, total protein 72 g/L, albumin 43 g/L, alkaline phosphatase (ALP) 47 U/L, alanine aminotransferase (ALT) 8 U/L, aspartate aminotransferase (AST) 11 U/L, total cholesterol 5.6 mmol/L, triglyceride 0.9 mmol/L, high-density lipoprotein cholesterol (HDL) 1.9 mmol/L, low-density lipoprotein cholesterol (LDL) 3.4 mmol/L, and HbA1c (%) 8.3% (Table 1). She had no evidence of retinopathy, microalbuminuria, or neuropathy.

**Table 1:** Clinical and laboratory measurements before GLP-1 therapy T1DM, type 1 diabetes mellitus; BMI, body mass index; HbA1c, glycated hemoglobin; eGFR, estimated glomerular filtration rate; ALP, alkaline phosphatase; ALT, alanine aminotransferase; AST, aspartate aminotransferase; HDL, high-density lipoprotein cholesterol; LDL, low-density lipoprotein cholesterol; GLP-1, glucagon-like peptide 1

Variables	Reference range	Patient with T1DM
Weight (kg)	-	63
BMI (kg/m^2^)	18.5-24.9	26.9
HbA1c(%)	<5.7	8.3
Creatinine (mg/dL)	44-80	0.71
eGFR (mL/min/1.73m^2^)	>90	>90
Bilirubin (mg/dL)	0-21	0.23
Total protein (g/L)	60-80	72
Albumin (g/L)	35-50	43
ALP (U/L)	35-104	47
ALT (U/L)	0-33	8
AST (U/L)	0-32	11
Total cholesterol (mmol/L)	<5.2	5.6
Triglycerides (mmol/L)	<1.7	0.9
HDL (mmol/L)	>1.0	1.9
LDL (mmol/L)	<2.59	3.4 mmol/L

Treatment

Treatment with once weekly subcutaneous semaglutide was initiated at a starting dose of 0.25 mg for two weeks, then 0.5 mg weekly for two weeks, followed by a maintenance dose of 1.0 mg weekly. The patient was educated about the pseudo-normoglycemic effect on CGM with semaglutide and was instructed to avoid excessively reducing her insulin dose to avoid the risk of diabetic ketoacidosis.

Outcome

Body Composition Analysis

**Figure 1:**
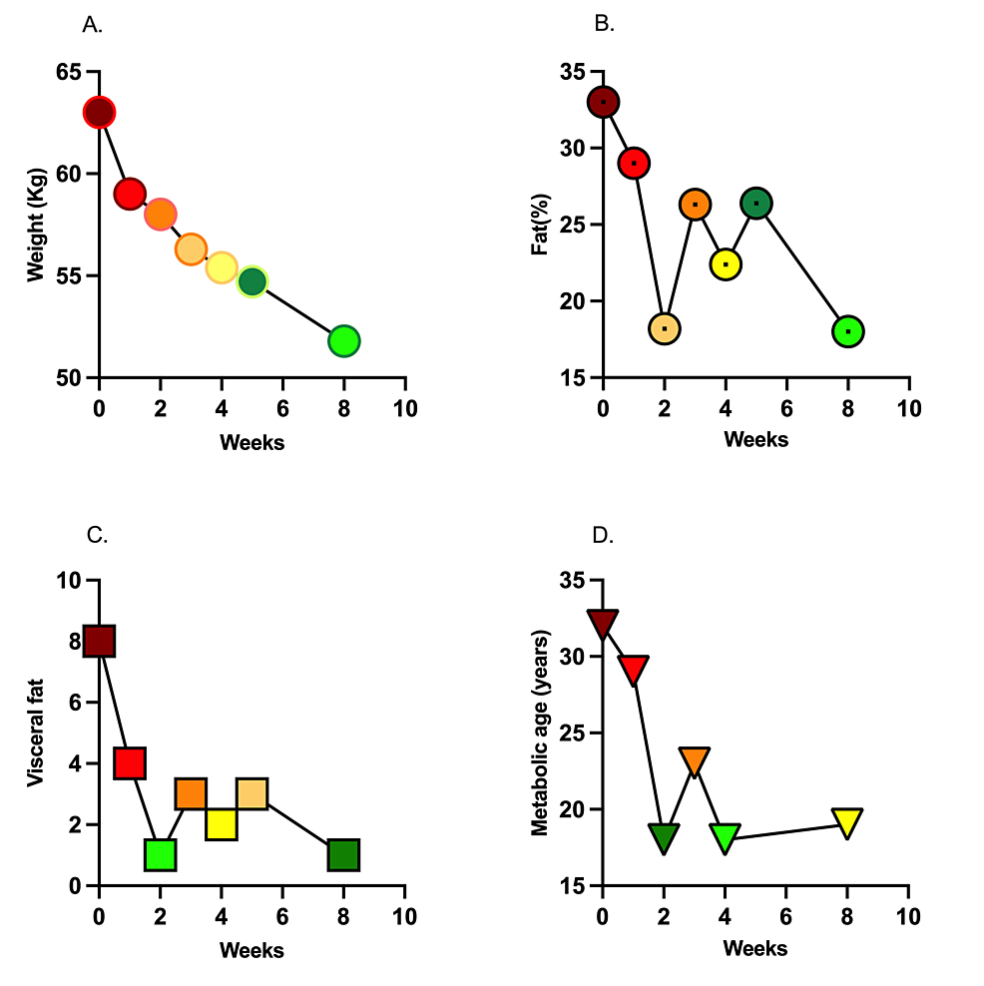
Body composition analysis using TANITA before and after once weekly dose of 1.0 mg subcutaneous semaglutide.

After two months of treatment with semaglutide, the patient’s weight had decreased to 51 kg (−12 kg) (Figure 1A), body fat percent was reduced to 18 (−15%) (Figure 1B), visceral fat was reduced to 1 (−7%) (Figure 1C), and metabolic age was reduced from 32 years to 19 years (Figure 1D).

Continuous Glucose Monitoring

**Figure 2:**
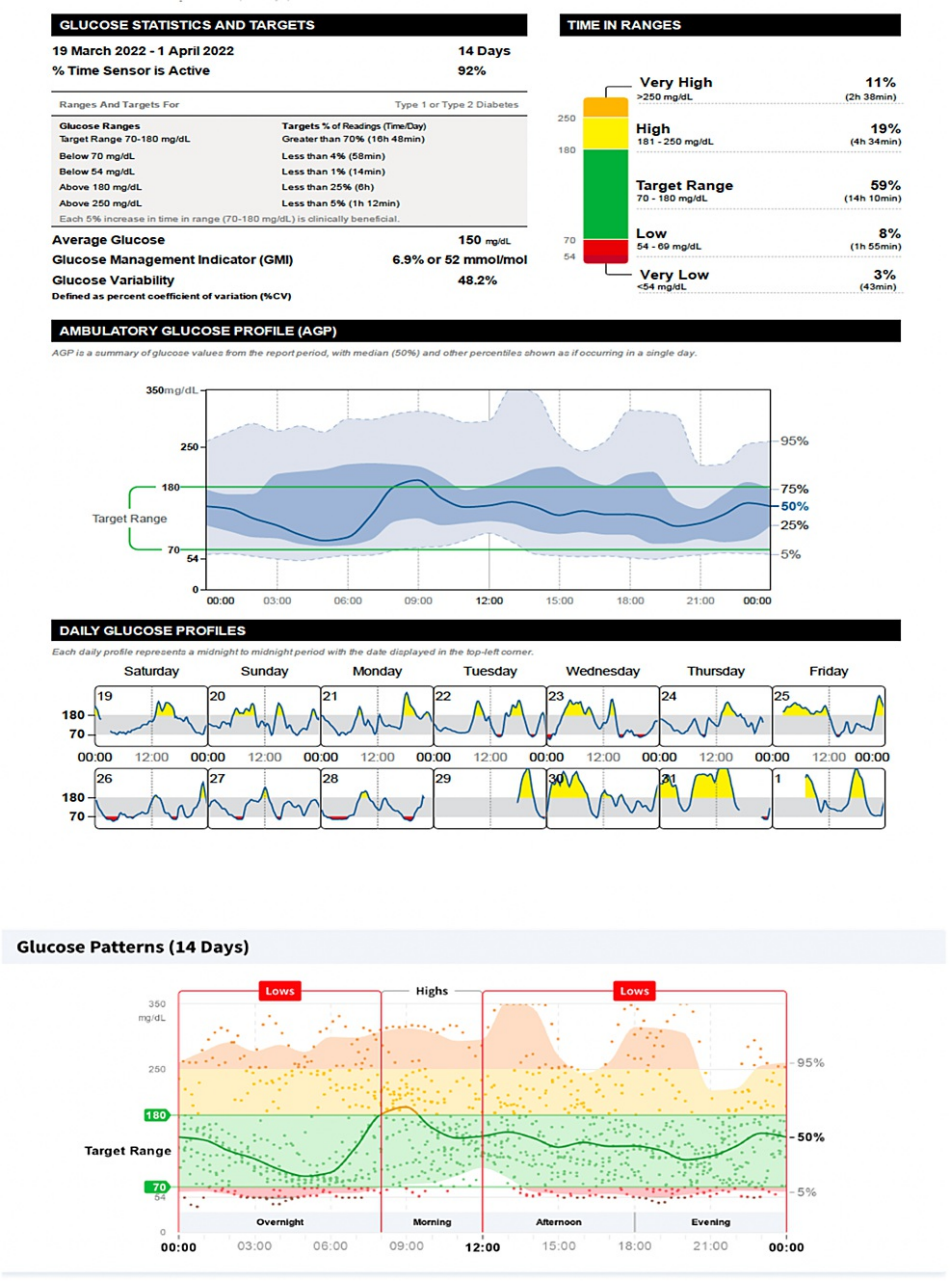
Ambulatory glucose profile before once weekly dose of 1.0 mg subcutaneous semaglutide. Glucose management indicator targets by the American Diabetes Association [5]

**Figure 3:**
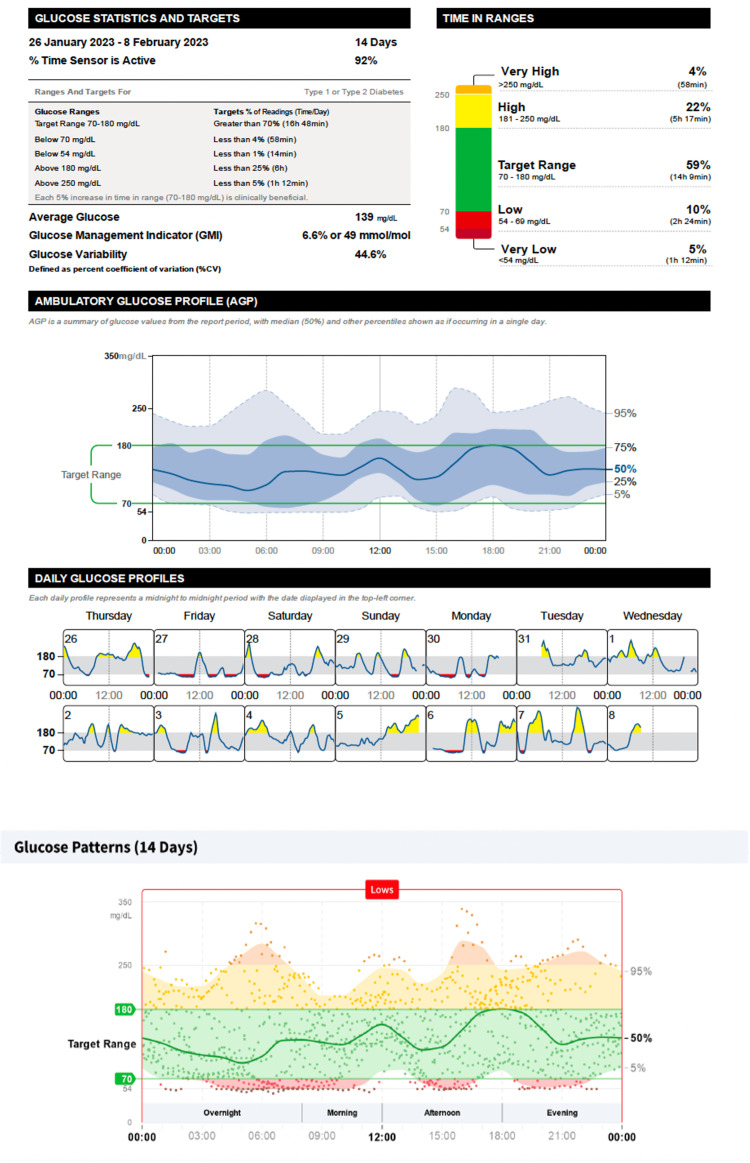
Ambulatory glucose profile after once weekly dose of 1.0 mg subcutaneous semaglutide. Glucose management indicator targets by the American Diabetes Association [5]

Time spent in level 2 hyperglycemia (time above range) was reduced from 11% (Figure 2) to 4% (Figure 3), with no change in time in range. An increase in time below range was associated with more hypoglycemic events from 19 to 21. Glucose management indicator was reduced from 6.9% to 6.6%, and glycemic variability was reduced from 48.2% to 44.6% (Figures 2, 3; Table 2). The dose of Glargine was reduced by 2U and that of Lispro insulin by ~2U based on carb counting.

**Table 2:** Glucose metrics before and after once weekly dose of 1.0 mg subcutaneous semaglutide. GMI, glucose management indicator; GV, glucose variability; TAR, time above range; TIR, time in range; TBR, time below range

Variables	Pre-semaglutide	Two months post-semaglutide
Average glucose (mg/dL)	150	139
GMI (%)	6.9	6.6
GV (%)	48.2	44.6
Time in range (%)		
TAR (>250 mg/dL): level 2 hyperglycemia	11	4
TAR (181-250 mg/dL): level 1 hyperglycemia	19	22
TIR (70-180 mg/dL)	59	59
TBR (54-69 mg/dL): level 1 hypoglycemia	8	10
TBR (<54 mg/dL): level 2 hypoglycemia	3	5

## Discussion

We have shown a dramatic improvement in weight and glucose variability with a negligible increase in the incidence of hypoglycemia after treatment with semaglutide in a woman with T1DM. Despite the introduction of new insulin analogues and CGM, strict glycemic control is difficult to achieve and maintain for most people with T1DM, especially as insulin is associated with an increased risk of hypoglycemic events and weight gain [6]. An increasing proportion of adults with T1DM are now overweight or obese, which impacts glycemic variability and overall glycemic control, which may increase the risk of both microvascular and macrovascular complications [7]. Increased glucose variability, driven by high and low glucose excursions, is associated with adverse vascular profiles in patients with T1DM [8]. A higher BMI in children and adolescents with T1DM is also associated with accelerated beta cell failure [9]. Adjunctive therapy with GLP-1RA alongside insulin may provide direct and indirect benefits by increasing glucagon and reducing insulin resistance in patients with T1DM [10]. Liraglutide, a short-acting GLP-1RA, previously showed favorable effects in patients with T1DM including weight loss, improvement in HbA1c, and lower insulin needs [10]. In a recent analysis of ADJUNCT ONE and ADJUNCT TWO, two randomized controlled phase 3 trials in T1DM, treatment with daily liraglutide 1.8 mg over 52 weeks was associated with a significant reduction in the placebo-adjusted HbA1c, body weight, and insulin dose (ADJUNCT ONE: −0.30%, −5.0 kg, and −12%; ADJUNCT TWO: −0.35%, −4.8 kg, and −10%, respectively) [6]. In a large retrospective cohort study of 1,822 patients with T1DM treated with predominantly short-acting GLP-1RAs, we have recently shown a clinically meaningful reduction in HbA1c, but with limited weight loss and cardiorenal benefits [11,12]. A recent study of patients with T1DM receiving low-dose semaglutide 0.5 mg weekly has shown improvement in weight and reduction in basal/bolus insulin [13]. We showed that semaglutide 1.0 mg leads to rapid and marked improvement in weight, percentage body fat and visceral fat, and metabolic age, but with an increased time below range, which warrants close monitoring for hypoglycemic episodes and insulin dose adjustment. Longer-term studies assessing the impact on microvascular and cardiorenal complications are required before adjunctive GLP-1 therapy can be recommended in patients with T1DM.

## Conclusions

Insulin is the first-line treatment in T1DM but is associated with weight gain, increased glycemic variability, and hypoglycemia. We showed that subcutaneous semaglutide once weekly is safe, well tolerated, and highly efficacious in enabling weight loss and improving glycemic variability and overall glycemic control in patients with T1DM. However, further research is required to establish the safety of adjunctive GLP-1 therapy in relation to the incidence of hypoglycemia and long-term complications in patients with T1DM.
